# Supplementary material for: High-Vigor Rootstock Exacerbates Herbaceous Notes in Vitis vinifera L. cv. Cabernet Sauvignon Berries and Wines Under Humid Climates
Source: Foods. 2025 Jul 31;14(15):2695. doi: 10.3390/foods14152695 (PMC12346354; doi:10.3390/foods14152695)
Supplement: Supplementary file 1 [file foods-14-02695-s001.zip › 3730876Supplemental Figure.pdf]

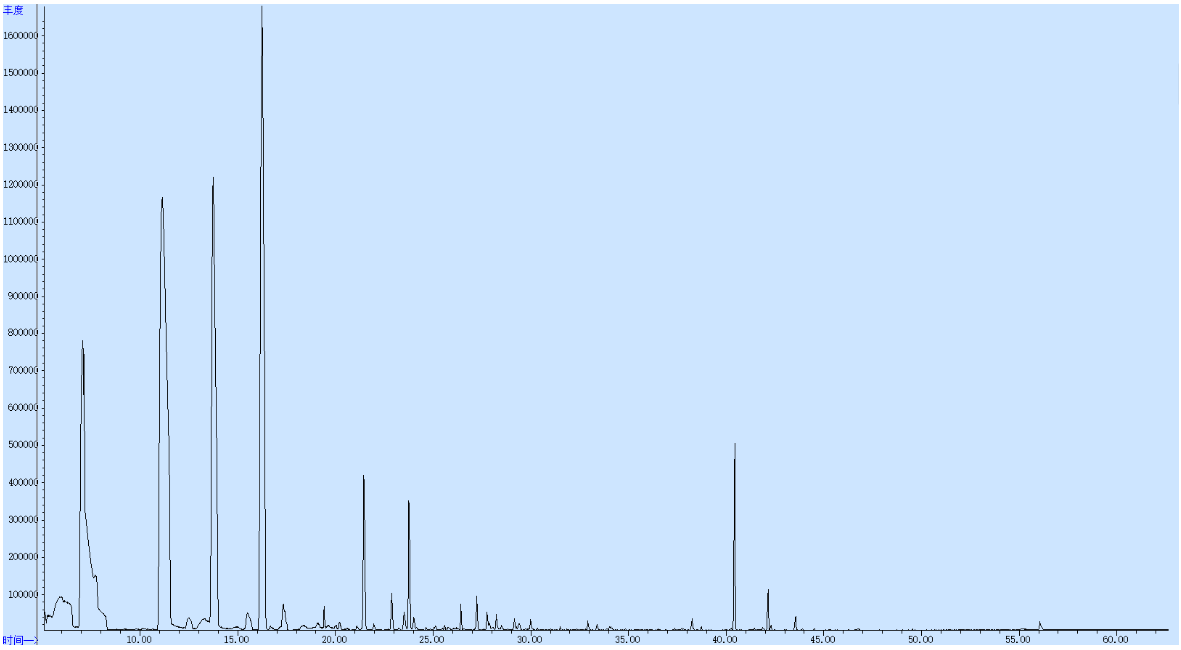

Supplementary Figure S1.Chromatogram of grape aroma compounds

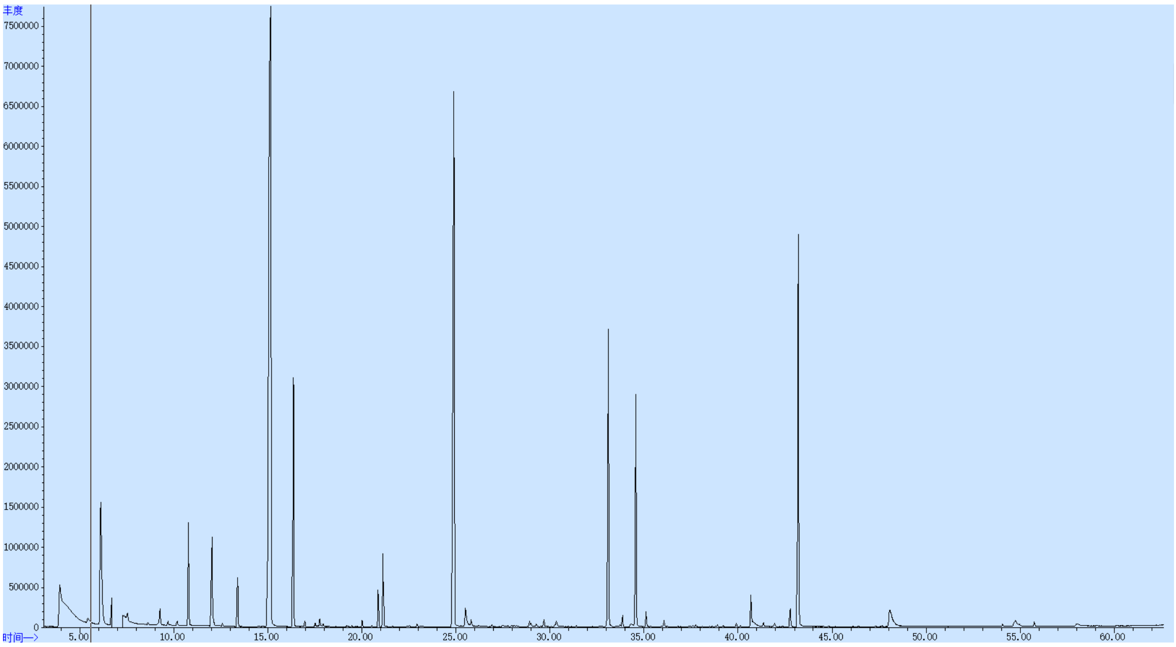

Supplementary Figure S2. Chromatogram of wine aroma compounds

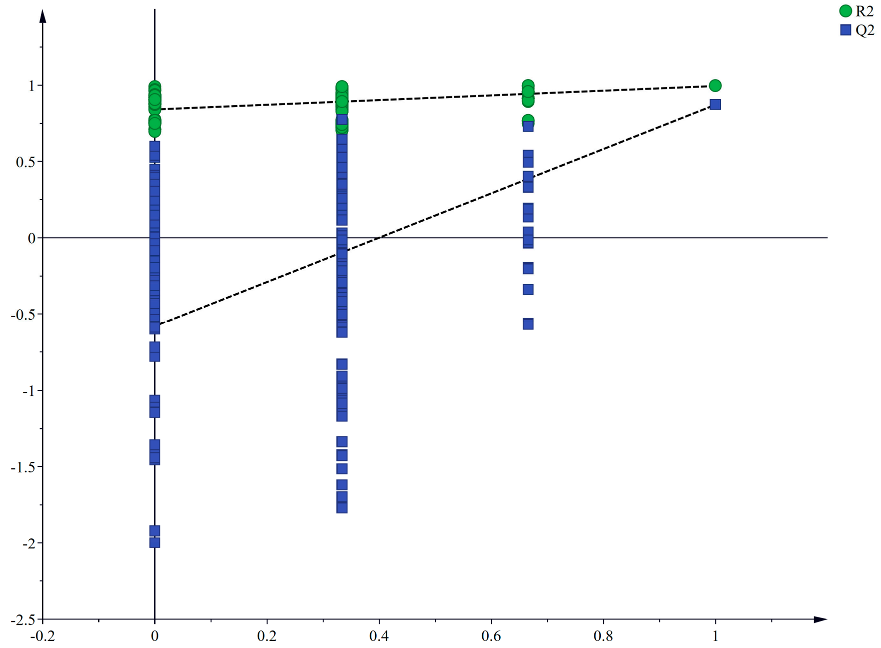

Supplementary Figure S3. 200 replacement tests based on CS/1103P grape aroma compound concentration

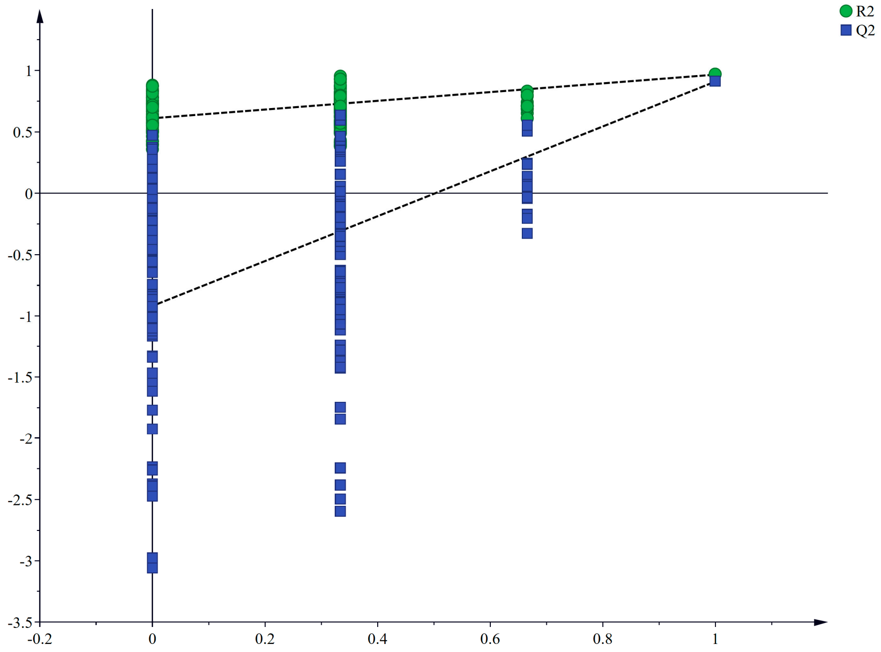

Supplementary Figure S4. 200 replacement tests based on CS/SO4 wine aroma compound concentration.

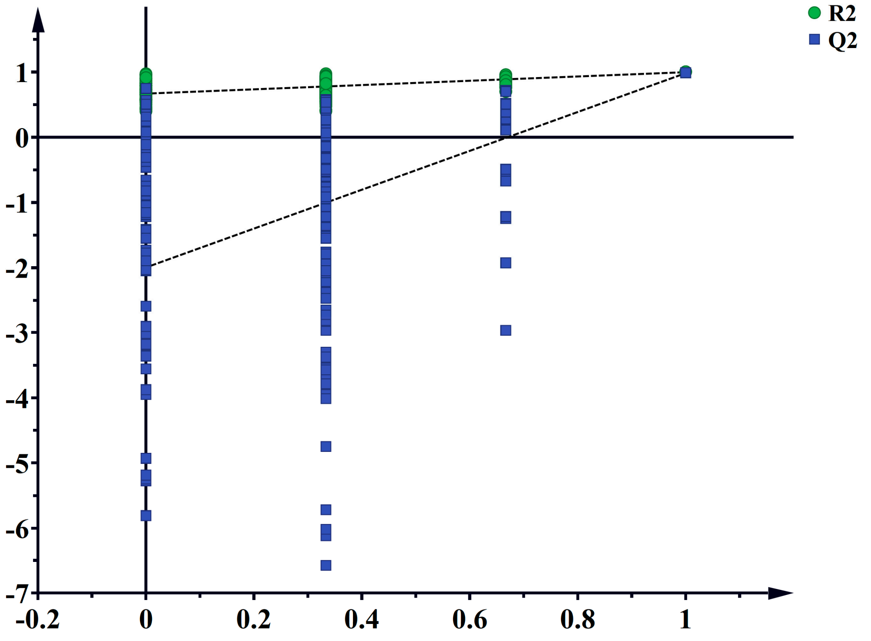

Supplementary Figure S5. 200 replacement tests based on CS/1103P wine aroma compound concentration.

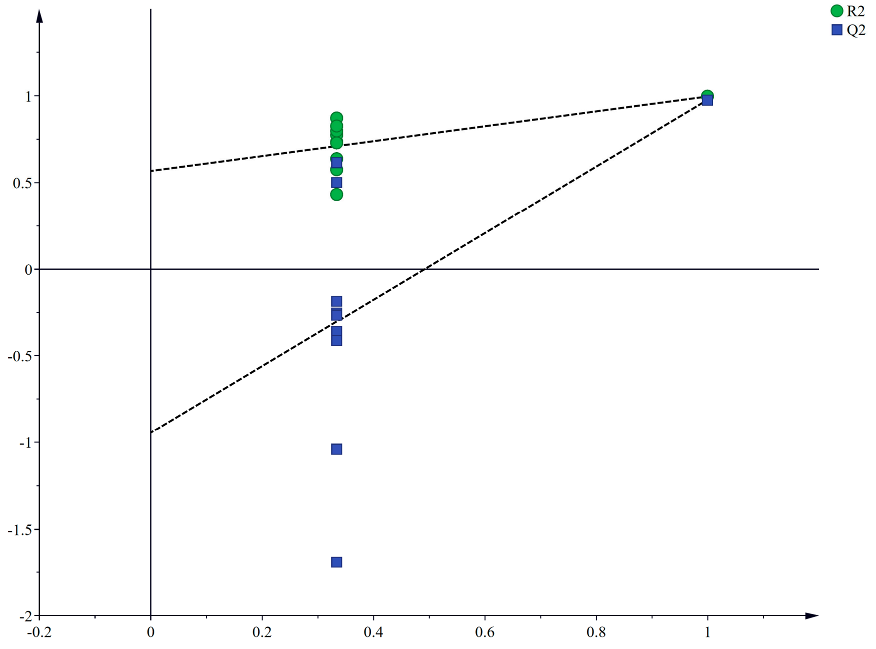

Supplementary Figure S6. 200 replacement tests based on CS/5A wine aroma compound concentration
